# Roxatidine attenuates mast cell-mediated allergic inflammation via inhibition of NF-κB and p38 MAPK activation

**DOI:** 10.1038/srep41721

**Published:** 2017-01-31

**Authors:** Minho Lee, Na Young Lee, Kyung-Sook Chung, Se-Yun Cheon, Kyung-Tae Lee, Hyo-Jin An

**Affiliations:** 1 Catholic Precision Medicine Research Center, College of Medicine, The Catholic University of Korea, 222, Banpo-daero, Seocho-gu, Seoul, 06591, Republic of Korea; 2 Department of Pharmacology, College of Korean Medicine, Sangji University, Gangwon-do 220-702, Republic of Korea; 3 Department of Pharmaceutical Biochemistry, Kyung Hee University, Seoul 130-701, Republic of Korea

## Abstract

Roxatidine is an active metabolite of roxatidine acetate hydrochloride which is a histamine H_2_-receptor antagonist that is used to treat gastric and duodenal ulcers. In this study, we investigated the anti-allergic inflammatory effects and the underlying molecular mechanism of roxatidine in phorbol 12-myristate 13-acetate and calcium ionophore (PMACI)-stimulated human mast cells-1 (HMC-1), compound 48/80-induced anaphylactic animal model and chemical allergen-induced contact hypersensitivity (CHS) models. Roxatidine suppressed the mRNA and protein expression of inflammatory cytokines such as TNF-α, IL-6, and IL-1β in PMACI-stimulated HMC-1 and compound 48/80-induced anaphylactic mice. In addition, roxatidine attenuated PMACI-induced nuclear translocation of NF-κB and the phosphorylation of MKK3/6 and MK2, which are both involved in the p38 MAPK pathway. Furthermore, we observed that roxatidine suppressed the activation of caspase-1, an IL-1β converting enzyme, in PMACI-stimulated HMC-1 and compound 48/80-induced anaphylactic mice. In CHS model, roxatidine significantly reduced ear swelling, increased number of mast cells, production levels of cytokines and migration of dendritic cells. Our findings provide evidence that the anti-allergic inflammatory properties of roxatidine are mediated by the inhibition of NF-κB and caspase-1 activation, p38 MAPK pathway and mast cell-derived cytokine production. Taken together, the *in vitro* and *in vivo* anti-allergic inflammatory effects suggest a possible therapeutic application of roxatidine in allergic inflammatory diseases.

Allergic disorders, such as anaphylaxis, hay fever, eczema and asthma, now afflict roughly 25% of people in the developed world. In allergic subjects, persistent or repetitive exposure to allergens, which typically are intrinsically innocuous substances common in the environment, results in chronic allergic inflammation^1^. Mast cells are central effector cells that cause immediate hypersensitivity and play multiple immunological roles in many inflammatory responses^2^. Immediate hypersensitivity is mediated by histamine release in response to the antigen cross-linking of immunoglobulin E (IgE) bound to high affinity surface receptors for IgE (FcεRI) on mast cells. Mast cells are activated by the process of degranulation, which triggers the release of mediators such as histamine by calcium signaling. The degranulation of mast cells can also be induced by the synthetic compound 48/80, phorbol 12-myristate 13-acetate (PMA), and calcium ionophore. Compound 48/80 has been used as a direct and convenient reagent to examine the mechanism underlying allergic reactions^3^.

NF-κB refers to a class of transcription factors involved in immune regulation, apoptosis, differentiation, inflammation, and cancer^4^. NF-κB is sequestered in the cytoplasm as an inactive complex bound by an inhibitor, known as IκB^5^. In response to a variety of signaling events, the IκB kinase complex (IKK) phosphorylates IκB proteins. This post-translational modification targets IκB for poly-ubiquitination and subsequent degradation by the 26 S proteasome^6^,^7^. The degradation of IκB proteins liberates NF-κB, allowing this transcription factor to translocate to the nucleus and activate its target genes. Besides regulation by IκB, NF-κB-dependent gene expression is also negatively regulated by the zinc finger protein A20, although the molecular mechanism remains unclear^8^. It has been reported that the activation of NF-κB is triggered by mitogen-activated protein kinases (MAPKs) such as extracellular signal-regulated kinase (ERK), c-Jun NH2-terminal kinase (JNK), and p38 MAPK^9^. However, other reports showed a negative regulation between NF-κB and MAPKs^10^. Therefore, the relationship between NF-κB and MAPKs is complex and appears to depend on the cell type and stimulus.

Roxatidine acetate hydrochloride (2-acetoxy-N-[3-[m-(1-piperidinylmethyl) phenoxy] propyl] acetamide hydrochloride) is a histamine H_2_-receptor antagonist that is used to treat gastric and duodenal ulcers^11^. This compound is rapidly converted to its active metabolite, roxatidine, by esterases in the small intestine, plasma, and liver. Thus, it cannot be found in plasma samples taken from volunteers after oral administration^12^. Roxatidine is used clinically as an anti-ulcer agent. This drug is also known to increase gastric mucus, inhibit gastric acid secretion, and ameliorate gastric mucosal injury caused by diclofenac or aspirin^13^,^14^. In particular, roxatidine has also been reported to suppress histamine release (thus inhibiting proton secretion) and inhibit the production of VEGF-1, an important marker of inflammation and angiogenesis^15^. In addition, we reported the anti-inflammatory activities of roxatidine including inhibition of NF-kB and p38 MAPK activation in LPS-induced RAW 264.7 macrophages^16^. Although roxatidine has been reported to show various bioactivities, the anti-allergic inflammatory effect of roxatidine remains unclear. Therefore, to evaluate the potential anti-allergic activity of compounds, we investigated the molecular mechanisms involved in the anti-allergic inflammatory properties of roxatidine in an activated human mast cells and in a murine model of anaphylactic shock and contact hypersensitivity (CHS).

## Results

### Roxatidine suppressed the PMACI-induced production of pro-inflammatory cytokines in HMC-1

**Figure 1:**
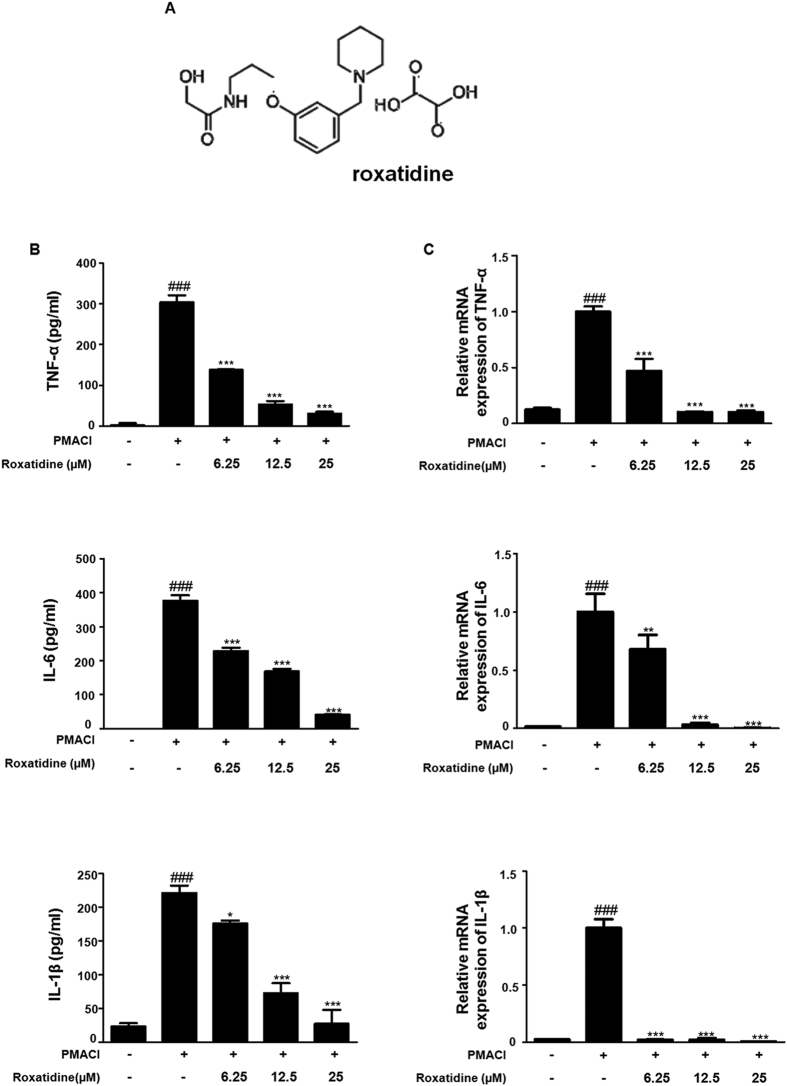
Effects of Roxatidine on PMACI-induced production of pro-inflammatory cytokines in HMC-1. (**A**) Chemical structure of roxatidine. (**B**) Cells were treated with 6.25,12.5 and 25 μM of roxatidine for 30 min prior to the addition of PMACI, and the cells were further incubated for 24 h. Cytokine production was measured by ELISA. (**C**) Cells were pre-treated with roxatidine for 30 min prior to the addition of PMACI for 6 h. The mRNA level of TNF-α, IL 6 and IL-1β was determined by qRT-PCR. Values represent mean ± S.D. of three independent experiments. ^*###*^*p* < *0*.*001* vs. the control group; *p < 0.05, **p < *0*.*01*, and ***p < *0*.*001* vs. PMACI-treated group.

To determine the inhibitory effects of roxatidine in pro-inflammatory cytokine production induced by PMACI, we investigated its effects on PMACI-induced TNF-α, IL-6, and IL-1β production (Fig. 1B) and their mRNA levels (Fig. 1C), by using EIA and qRT-PCR, respectively. Pretreatment with roxatidine down-regulated the PMACI-induced TNF-α, IL-6, and IL-1β production and their mRNA expression in a dose-dependent manner. These data indicated that roxatidine regulated the PMACI-induced expression of TNF-α, IL-6, and IL-1β through transcriptional inhibition. In addition, these inhibitory effects of roxatidine were not caused by nonspecific cytotoxicity, as roxatidine had no effect on cell viability at concentrations from 6.25 to 100 μM as determined by MTT assay (Supplementary Fig. 1).

### Roxatidine suppressed PMACI-induced NF-κB and caspase-1 activation in HMC-1

**Figure 2:**
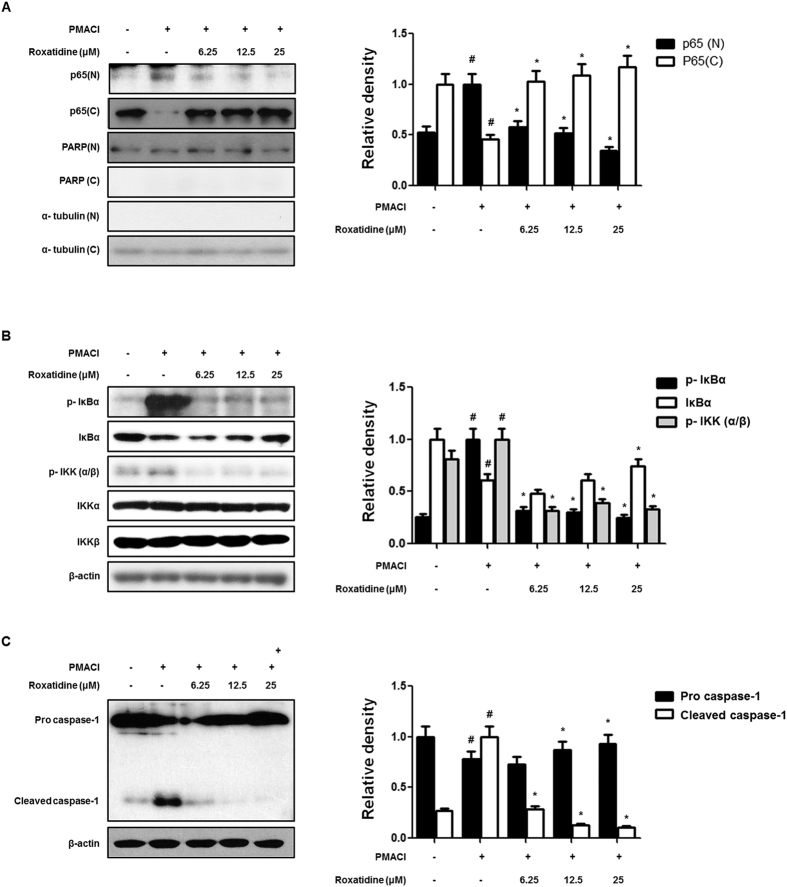
Effects of roxatidine on PMACI-induced NF-κB and caspase-1 activation in HMC-1. (**A**) Cells were pre-treated with roxatidine for 30 min prior to the addition of PMACI for 30 min. Nuclear (N) and cytosolic (**C**) extracts were isolated and the levels of p65 in each fraction were determined by western blot analysis. PARP and α- tubulin were used as internal controls. (**B** and **C**) Cells were pre-treated with roxatidine for 30 min prior to the addition of PMACI for 15 min. Nuclear and cytosolic extract and total proteins were prepared, and western blot analysis was performed by using specific antibodies. Densitometric analysis was performed using Bio-Rad Quantity One Software. The data shown represent mean ± SD of three independent experiments. ^*#*^*p* < *0*.*05* vs. the control group; *p < 0.05 vs. PMACI-treated group.

Because the activation of NF-κB is critically required for the transcriptional regulation of inflammation^4^, we examined the effect of roxatidine on PMACI-stimulated nuclear translocation of NF-κB. It was found that roxatidine markedly suppressed the PMACI-stimulated nuclear translocations of p65 subunit of NF-κB in HMC-1 (Fig. 2A). In its inactive state NF-κB binds to its inhibitor protein, IκB-α, in the cytoplasm. After cellular stimulation IκB-α is phosphorylated at specific serine residues and undergoes polyubiquitination and proteasomal degradation, which free NF-κB, allowing it to be translocated to the nucleus^17^. Thus, we explored whether roxatidine inhibits the PMACI-induced phosphorylation and degradation of IκB-α in HMC-1. As shown in Fig. 2B, the PMACI-induced phosphorylation and degradation of IκB-α were significantly blocked by roxatidine pretreatment. In addition, because IKK-α/β are upstream kinases of IκB-α in the NF-κB signal pathway, we examined the effects of roxatidine on the PMACI-induced activation of IKK-α/β in HMC-1. We found that roxatidine markedly reduced PMACI-induced IKK-α/β phosphorylation, but did not affect the total amounts of IKK-α/β.

Caspase-1 contributes to inflammation by two distinct pathways: proteolysis of pro-IL-1β, and RIP2-dependent activation of NF-κB and p38 MAPK mediated by the caspase recruitment domain^18^. Thus, we investigated whether roxatidine prevents PMACI-induced caspase-1 activation. As shown in Fig. 2C, we found that treatment with roxatidine resulted in an increase in protein levels of procaspase-1 and the disappearance of the corresponding cleaved bands in PMACI-induced HMC-1. These data indicated that the activation of caspase-1 may be involved in the roxatidine-mediated inhibition of PMACI-induced allergic inflammatory responses in HMC-1.

### Roxatidine inhibited PMACI-induced activation of p38 MAPK in HMC-1

**Figure 3:**
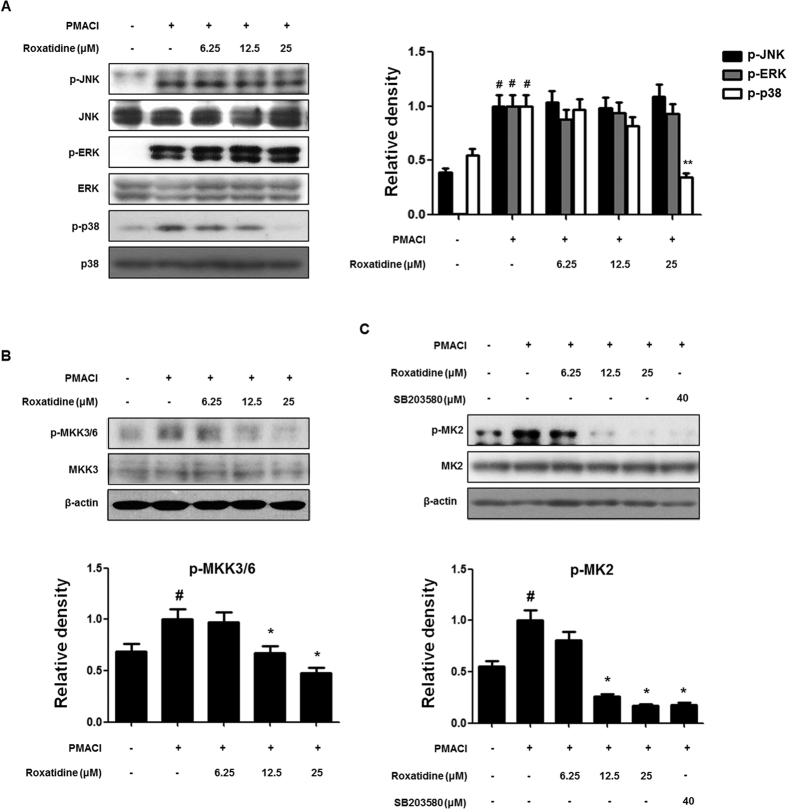
Effects of roxatidine on PMACI-induced activation of MAPKs MKK3/6 and MK2 in HMC-1. (**A** and **B**) Cells were pre-treated with roxatidine for 30 min prior to the addition of PMACI for 15 min. (**C**) Cells were pre-treated with roxatidine or SB203580 (40 μM) for 30 min prior to the addition of PMACI for 15 min. Total proteins were prepared, and western blot analysis was performed using specific antibodies. Densitometric analysis was performed using Bio-Rad Quantity One Software. The data shown represent mean ± SD of three independent experiments. ^*#*^*p* < *0*.*05* vs. the control group; *p < 0.05 and **p < *0*.*01* vs. PMACI-treated group.

MAPKs, including p38, JNK, and ERK1/2, are involved in the signal transduction pathways that lead to the regulation of inflammatory mediators. Moreover, the activation of NF-κB is mediated mainly via a group of proteins referred to as MAPKs^19^. Therefore, we investigated whether the inhibition of allergic inflammatory responses by roxatidine are mediated through the MAPK pathway in PMACI-stimulated HMC-1 by western blot analysis. As shown in Fig. 3A, PMACI significantly induced the phosphorylation of ERK 1/2, JNK, and p38 MAPK within 15 min, and roxatidine pretreatment suppressed the PMACI-induced activation of p38 MAPK, but did not affect the phosphorylation of ERK or JNK. Furthermore, total ERK 1/2, JNK, and p38 MAPK levels were unaffected by PMACI or by PMACI plus roxatidine.

MKK3 is a protein kinase that phosphorylates and activates p38 MAPK but does not phosphorylate the related JNK or ERK MAPKs^20^. MKK3 is therefore a specific activator of p38 MAPK that is independent of the JNK and ERK signaling pathways. To investigate the kinases upstream of p38 MAPK, we examined whether roxatidine prevents the PMACI-induced phosphorylation of MKK3/6. It was found that cells pretreated with roxatidine had significantly suppressed phosphorylation compared with those treated with PMACI alone, but total amounts of MKK3/6 were unaffected (Fig. 3B). MK2 is a downstream target of p38 MAPK and is required for inflammation^21^. Upon stress-stimulation, a subpopulation of activated p38 and MK2 form a complex in the nucleus of the cell, and p38 and MK2 are co-exported to the cytoplasm^22^. The cytoplasmic location of activated MK2 corresponds with its role in regulating translation. Therefore we examined the effects of roxatidine on the p38 MAPK/MK2 signaling pathway to verify whetherit is involved in the phosphorylation of p38 MAPK induced by PMACI (Fig. 3C). It was found that roxatidine significantly inhibited the PMACI-induced phosphorylation of MK2. For further confirmation, we examined the inhibitory effects of specific inhibitors for p38 MAPK (SB203580) using western blot analysis.

### Roxatidine ameliorated compound 48/80-induced allergic hypersensitivity in anaphylactic animal model

**Figure 4:**
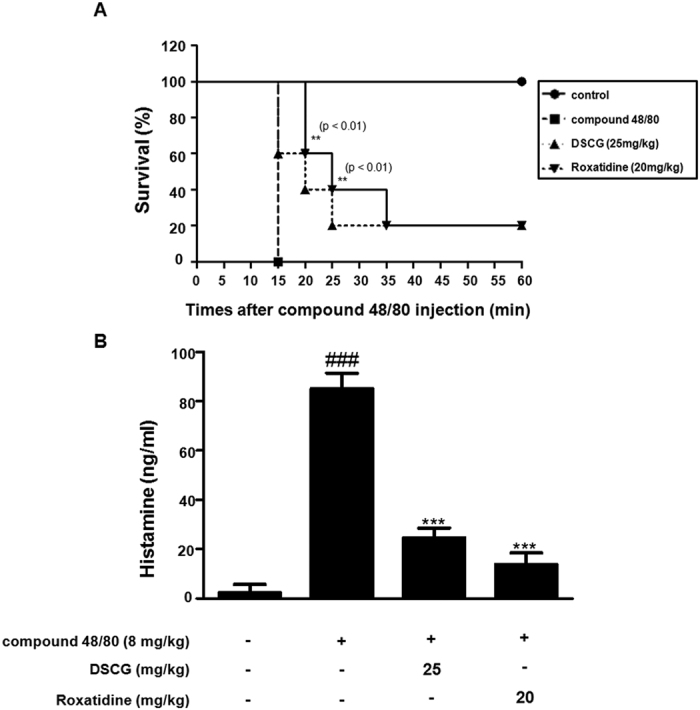
Effects of roxatidine on compound 48/80-induced mortality rate and histamine release in anaphylactic animal models. Mice were administrated with roxatidine (20 mg/kg, p.o.), DSCG (25 mg/kg, p.o) and PBS as a vehicle (n = 6 per group) for 1 h before compound 48/80 injection (8 mg/kg i.p.). (**A**) Survival rates of these mice were monitored for 1 h. (**B**) Histamine concentrations were measured in culture medium by EIA. Densitometric analysis was performed using Bio-Rad Quantity One Software. The data shown represent mean ± SD of three independent experiments. ^*###*^*p* < *0*.*001* vs. the control group; **p < *0*.*01* and ***p < *0*.*001* vs. PMACI-treated group.

To assess the anti-allergic inflammatory effect of roxatidine *in vivo*, we investigated its effect on the survival rate of mice with compound 48/80-induced hypersensitive anaphylaxis. After the intraperitoneal injection of compound 48/80, all mice were monitored for 1 h and the survival rate was determined. As shown in Fig. 4A, all mice died with within 15 min after compound 48/80 injection, whereas DSCG (25 mg/kg, *p*.*o*) and roxatidine (20 mg/kg, *p*.*o*) increased the survival rate to 20% at 60 min after compound 48/80 injection. Interestingly, roxatidine is might be more effect than DSCG in delaying the lethality rate in anaphylactic animal model. The results of the lethality test may correlate with the measured serum histamine levels. Among the performed and newly synthesized inflammatory substances released on degranulation of mast cells, histamine is the best characterized and most potent vasoactive mediator implicated in the acute phase of immediate allergic reactions^23^. Thus, to determine whether roxatidine inhibits histamine release from mast cells, we determined the serum level of histamine after compound-40/80-induced anaphylactic shock. Roxatidine at a dose of 20 mg/kg decreased the compound 48/80-induced serum histamine levels (Fig. 4B). These results indicate that roxatidine provided potential protection against anti-allergic inflammation via the inhibition of histamine release during the systemic anaphylactic reaction.

### Roxatidine suppressed compound 48/80-induced allergic inflammation in anaphylactic animal model

**Figure 5:**
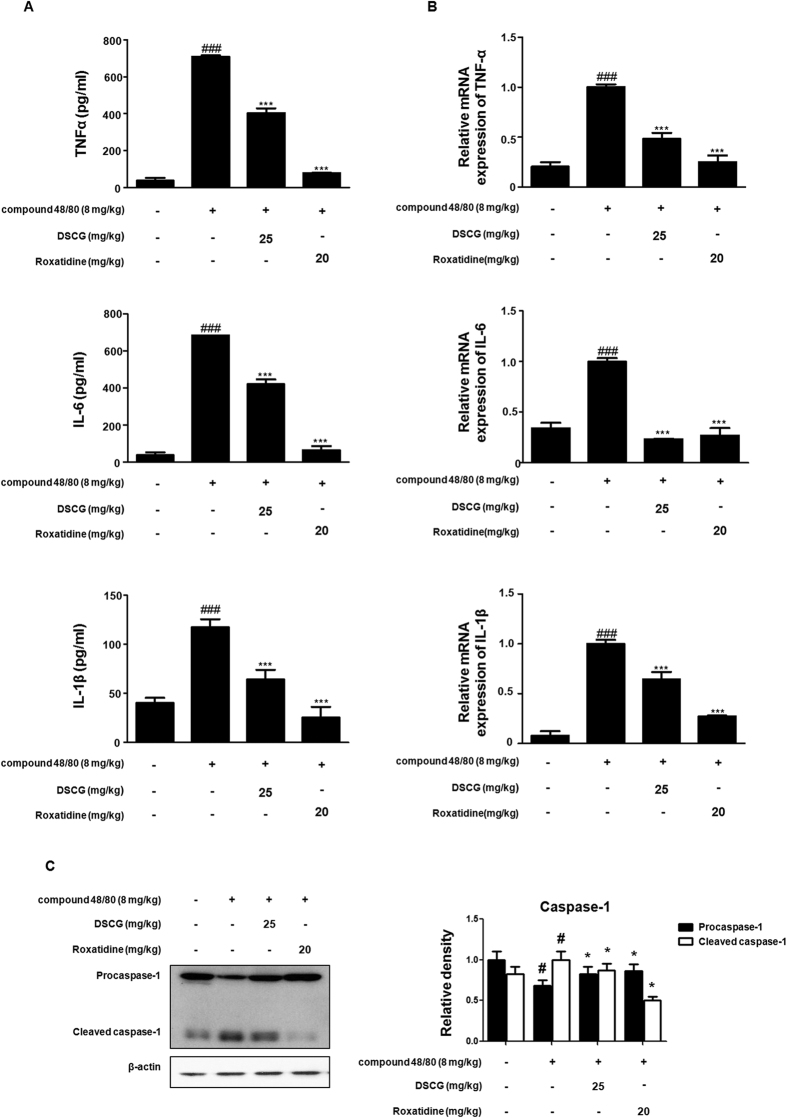
Effects of roxatidine on compound 48/80-induced cytokines and caspase-1 activation in anaphylactic animal models. Mice were administrated with roxatidine (20 mg/kg, p.o.), DSCG (25 mg/kg, p.o) and PBS as a vehicle (n = 6 per group) for 1 h before compound 48/80 injection (8 mg/kg i.p.). (**A**) Serum level of TNF-α, IL-6 and IL-1β were determined by using EIA kits. (**B**) Total RNA prepared from the liver tissue and the level of TNF-α, IL-6 and IL-1β were determined by quantitative real-time PCR. (**C**) Expression of procaspae-1 was determined by western blot analysis using specific antibodies. Densitometric analysis was performed using Bio-Rad Quantity One Software. The data shown represent mean ± SD of three independent experiments. ^*#*^*p* < *0*.*05* and ^*###*^*p* < *0*.*001* vs. the control group; *p < *0*.*05* and ***p < *0*.*001* vs. PMACI-treated group.

Mast cells are activated by PMACI and secrete inflammatory mediators, including a variety of cytokines^23^. To evaluate levels of cytokines in the allergic reaction, we examined the production and mRNA levels of cytokines in anaphylactic mice. As shown in Fig. 5A, compound 48/80 administration markedly increased the production and mRNA levels of TNF-α, IL-6, and IL-1β, but pre-treatment with roxatidine (20 mg/kg, p.o.) for 1 h before compound 48/80 administration significantly decreased these pro-inflammatory cytokines. Furthermore, we investigated the effect of roxatidine on the activation of caspase-1. Administration of roxatidine inhibited the compound 48/80-induced degradation of procaspase-1 and appearance of the corresponding cleaved bands, indicating the activation of caspase-1 in anaphylactic mice. These results suggest that the suppressive effects of roxatidine on NF-κB-regulated gene transcription are responsible for its anti-inflammatory effect via the regulation of the NF-κB and caspase-1 activation in this animal model of anaphylactic shock.

### Roxatidine affected the sensitization phase in CHS-induced animal model

**Figure 6:**
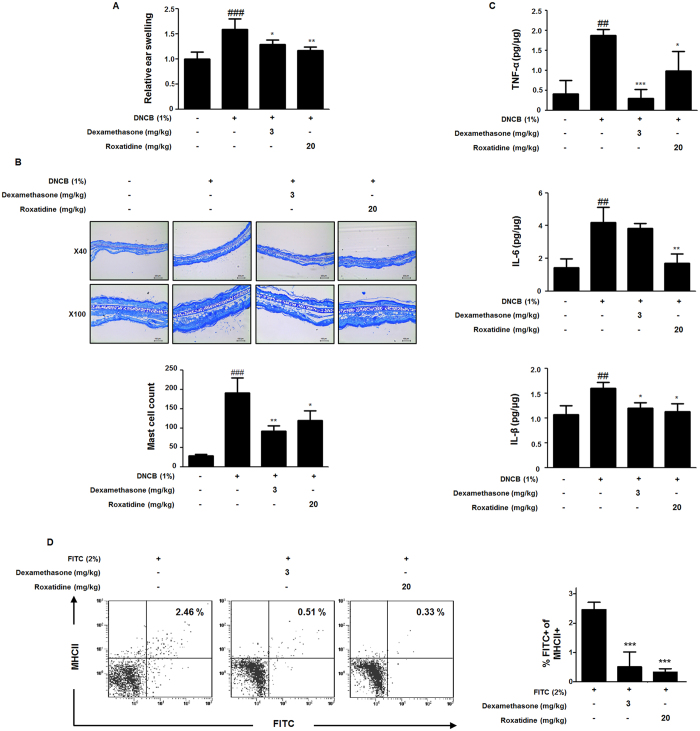
Effects of roxatidine on the sensitization phase in CHS. (**A**) Mice were administrated with roxatidine (20 mg/kg, p.o.), dexamethasone (3 mg/kg, p.o.) and a vehicle (n = 6 per group) for 1 h before 1% DNCB-induced sensitization. Relative ear swelling was measured as the increases compared to prechallenge ear weight. (**B**) Sections were stained with toluidine blue to identify mast cells and mast cells were counted with a microscope at a magnification of 40× (scale bar: 250 μm) and 100× (scale bar: 100 μm), respectively. (**C**) The cytokine levels of TNF-α, IL-6 and IL-1β from the ear tissue were determined by EIA kits. (**D**) Mice were administrated with roxatidine, dexamethasone and a vehicle (n = 6 per group) for 1 h before 2% FITC-induced sensitization. The number of FITC^+^ MHC classII^+^ DCs in the draining LNs of mice 48 h after sensitization of FITC. The data shown represent mean ± SD of three independent experiments. ^*##*^*p* < *0*.*01* and ^*###*^*p* < *0*.*001* vs. the control group; *p < *0*.*05*, **p < *0*.*01* and ***p < *0*.*001* vs. CHS-induced group.

To investigated the role of roxatidine on mast cell-related cutaneous acquired immune response, we used DNCB-induced CHS as a model. As shown in Fig. 6A, DNCB treatment significantly increased the the relative ear swelling ratio, but pre-treatment with roxatidine (20 mg/kg, p.o.) and dexamethasone (3 mg/kg, p.o.) for 1 h before DNCB treatment significantly decreased these ratios. Moreover, in order to evaluate whether inflammatory cells had infiltrated the skin after DNCB treatment, sections of ear tissue were stained with toluidine blue to detect mast cells. The number of mast cells in the ear markedly increased in DNCB-treated group compared with control mice, whereas treatment with roxatidine (20 mg/kg, p.o.) and dexamethasone (3 mg/kg, p.o.) significantly suppressed the number of mast cells (Fig. 6B). In addition to, DNCB treatment markedly increased the production levels of TNF-α, IL-6, and IL-1β, but pre-treatment with roxatidine (20 mg/kg, p.o.) and dexamethasone (3 mg/kg, p.o.) for 1 h before DNCB treatment significantly decreased these pro-inflammatory cytokines (Fig. 6C).

An essential step in the sensitization phase for CHS is the migration of chemical allergen-bearing cutaneous dendritic cells (DCs) such as epidermal Langerahans cells (LCs) and immature dermal DCs, into skin-draining lymph nodes (LNs)^24^. In line this report, to investigate the effect of roxatidine on the action of mast cells in the sensitization phase, we determined the migration of FITC-bearing DCs that have an opportunity to interact with mast cells present in LNs. As shown in Fig. 6D, in FITC-induced cutaneous DCs migration model, we found that the number of FITC^+^ MHC class II^+^ DCs in the draining LNs 48 h after FITC application was significantly attenuated with pre-treatment of roxatidine (20 mg/kg, p.o.) and dexamethasone (3 mg/kg, p.o.). Our findings suggested that roxatidine showed allergic inflammatory response involving cytokine-mediated mast cell activation in sensitization phase of CHS model.

## Discussion

Mast cells play key roles in allergic inflammation, as they are the effector cells of the immediate IgE-driven hypersensitivity reaction^25^. During this reaction, the body is exposed to the allergen, which cross-links two specific IgE molecules presented on FcεRI on the cell membrane of mast cells. This initiates an activation cascade resulting in calcium mobilization, degranulation of histamine and other inflammatory mediators, and synthesis of cytokines^26^. Proinflammatory cytokines such as TNF-α, IL-6, IL-1β, and IL-8 promote inflammation, leukocyte infiltration, granuloma formation, and tissue fibrosis, and are thought to be initiators of cytokine related inflammation states by stimulating cytokine production^27^. TNF-α is secreted by mast cells, macrophages, and T cells in the allergic inflammatory signaling. TNF-α induces the expression of vascular endothelial cell adhesion factors; it accumulates leukocytes as a result of the inflammatory response^28^,^29^. IL-6 is a chronic inflammatory cytokine, causing T-cell activation and IgE production^30^. The maturation of pro-IL-1β occurs in a large caspase-1 containing protein complex known as the inflammasome^31^. After maturation, these cytokines are released or secreted with mechanisms that have not yet been identified. An autocrine or paracrine action of IL-1β may lead to the activation of NF-κB^18^. These reports indicate that reduction of proinflammatory cytokines from mast cells is one of the key indicators of reduced inflammatory symptoms. In the present study, roxatidine decreased mRNA levels of TNF-α, IL-6, and IL-1β in PMACI-stimulated HMC-1 and the serum of anaphylactic shock animal models. These data indicated that roxatidine shows an anti-allergic inflammatory effect by suppressing proinflammatory cytokines *in vitro* and *in vivo*.

The expression of proinflammatory cytokines is dependent from mast cells in the activation of the transcription factor NF-κB^5^. The activation of NF-κB results in the phosphorylation, ubiquitination, and proteasome-mediated degradation of IκB, and the subsequent translocation of NF-kB to the nucleus^32^. Furthermore, IKK complex activation is required for IκBα ubiquitination, which phosphorylates IkBa at Ser 32 and Ser 36 in the NF-κB translocation dependent pathway. In this study, roxatidine was found to suppress PMACI-induced degradation or phosphorylation of IκBα. In addition, we also found that roxatidine reduced the PMACI-induced phosphorylation of IKK, indicating disturbance of IKK activation, whereas roxatidine did not affect the PMACI-induced total IKKα or IKKβ levels.

It has been suggested that NF-κB activation is mediated by the phosphorylation of MAPK family members, such as, ERK, JNK, and p38 MAPK^33^. MAPK family members are known to play a central role in cytokine production, although the selective involvement of each member appears to rely on the cell type and the nature of the stimulus. In mast cells that are major sources of cytokines during IgE-dependent reaction, it has been reported that aggregation of FcεRI activates several sequential protein kinase pathways, leading to increased activity of the MAPK family^34^. In particular, the p38 MAPK pathway is required for the production and activity of multiple pro-inflammatory cytokines via NF-κB activation^35^. We examined the effects of roxatidine on the PMACI-induced phosphorylation of MAPK in HMC-1. We found that roxatidine prevented the PMACI-induced phosphorylation of p38 MAPK, but that it had no effect on the phosphorylation of ERK and JNK. To confirm whether roxatidine had inhibitory effects on PMACI-induced p38 MAPK signaling, we examined whether roxatidine prevents the PMACI-induced phosphorylation of MKK3/6 and MK2. It was found that cells pretreated with roxatidine had markedly suppressed phosphorylation compared with those treated with PMACI alone, but that this did not affect total amounts of MKK3/6 and MK2. Therefore, these data suggest that inhibition of the signaling cascade involving NF-κB and p38 MAPK by roxatidine can lead to suppression of inflammatory gene expression in PMACI-induced HMC-1. In RAW 264.7 macrophages, roxatidine also ameliorates inflammatory states through inhibition of MK2 and p38 phosphorylation, but not JNK and ERK MAPK phosphorylation^16^. This indicates that roxatidine works with the same mechanism in different immune cell lines.

The caspase family is comprised of 13 different cysteine proteases that are mainly involved in the apoptotic pathway^36^. Among them, caspase-1 exists in a catalytically inactive 45 kDa proenzyme and is activated by cleavage into two non-identical subunits, p20 and p10, which is less involved in the apoptotic cascade but is prominent in inflammation because of its pivotal role in regulating the cellular export of proinflammatory cytokines such as IL-1β. Caspase-1 is elevated in intestinal macrophages during inflammatory bowel disease^37^ and in a variety of organs, including the brain, in response to bacterial LPS administration^38^. Further evidence of the role played by caspase-1 in inflammation comes from studies on caspase-1-deficient (^−/−^) mice and caspase-1 pharmacological inhibitors. Caspase-1^−/−^ mice display an alteration in the export of several proinflammatory cytokines, namely IL-1β, IL-1α, IL-6, and TNF-α, although IL-1α, IL-6, and TNF-α are not substrates for caspase-1^39^. The activation of caspase-1 is also involved in the NF-κB signaling pathway^18^. These studies indicated that the activation of caspase-1 is an attractive target for treatment of inflammatory disease. We demonstrated that the inhibitory effects of roxatidine on production of inflammatory mediators might be the result of the regulation of caspase-1 activation in PMACI-stimulated HMC-1 and compound 48/80-induced anaphylactic animal models.

Histamine is considered a feature arbitration of mast cells^26^. Histamine is a biogenic amine synthesized from _L_-histidine by histidine decarboxylase and stored within granules mainly in basophils and mast cells^40^. The actions of histamine are considered to be mediated by at least two distinct classes of receptors, the H_1_- and H_2_-receptors, which are widely distributed throughout the body^41^. They evoke different responses in different cell types. Responses mediated via H_2_-receptors seem to involve the adenylate cyclase system^42^, and changes in intracellular concentrations of cAMP and cGMP occur early in the mitogenic reaction studied^43^. Several antihistamines, namely the H_1_-blockers azelastine, loratadine, and cetirizine, as well as the H_2_-receptor antagonist ranitidine, inhibited cytokine production and secretion from mast cells and basophils^44^. Roxatidine also acts as a competitive H2 receptor antagonist that is used to treat gastric and duodenal ulcers, and is known to suppress inflammatory responses via inhibition of NF-κB activation in macrophages^16^. In this study, we observed that proinflammatory cytokine productions and histamine release were inhibited by roxatidine in compound 48/80-mediated anaphylactic mice. From that result, we postulate that roxatidine presents a possible application in allergic inflammatory diseases.

It is well known that mast cell activation induces production of mediator such as cytokines and chemokines^45^. Mast cell-produced cytokines and chemokines act both to activate local cells and to promote cell recruitment to sites of inflammation. For example, mast cell-derived TNF has been shown to promote DC activation and antigen presentation^46^. CHS is a chemical allergen- (i.e., hapten such as FITC)-induced T cell-dominated delayed-type hypersensitivity reaction reflecting the allergic contact dermatitis^47^. In mice exposed epicutaneously to high levels of FITC, some FITC can enter the lymphatic or systemic circulation and thereby reach DCs already present in the LNs^48^. According to our data, administration of roxatidine (20 mg/kg, p.o.) significantly suppressed DNCB-induced ear swelling, increased number of mast cells and production levels of cytokines. Furthermore, administration of roxatidine (20 mg/kg, p.o.) inhibited migration of FITC^+^ MHC class II^+^ DCs into skin-draining LNs in FITC-induced CHS model. These results suggest that roxatidine ameliorates chemical allergen-induced CHS by inhibiting cytokine-mediated mast cell activation and migration of DCs.

In conclusion, our results suggest that roxatidine inhibits PMACI-mediated production of proinflammatory cytokines and these inhibitions by roxatidine appears to be involved in suppression of NF-κB and caspase-1 activation in HMC-1. In addition, roxatidine affected the phosphorylation of MKK3/6 and MK2, suggesting that the inhibitory effect of roxatidine on pro-inflammatory cytokines is mediated not only via NF-κB activation but also p38 MAPK. Moreover, roxatidine ameliorates compound 48/80-induced anaphylactic mice and chemical allergen-induced CHS mice. These findings indicate that the biological properties of roxatidine can be used as potential candidates for pharmacologic intervention using bio-therapeutic inhibitors for treating allergic inflammatory diseases.

## Methods

### Chemicals and reagents

For this study, roxatidine (≥98% purity, Fig. 1A), primary antibodies for p65, p–IκBα, IκBα, p-ERK, p-p38, p-JNK, ERK, p38, JNK, PARP, α-tubulin, caspase-1, MKK3/6, MAPK-activated protein kinase 2 (MK2), and β-actin and peroxidase-conjugated secondary antibodies were purchased from Santa Cruz Biotechnology, Inc. (Santa Cruz, CA, USA). p-IKK, IKK, p-Akt, Akt, p-MKK3/6, and p-MK2 primary antibodies were obtained from Cell Signaling Technology (Danvers, MA) and M5/114.15.2 (MHC class II) antibody was purchased from eBioscience (eBioscience, Affimatrix Phorbol 12-myristate 13-acetate (PMA), calciumionophore A23187 (Calcymycin; C29H37N3O6), 3-(4,5-Dimethylthiazol-2-yl)-2,5-diphenyl tertazolium bromide (MTT), fluorescein isothiocyanate (FITC) isomer-I, 2,4-dinitrochlorobenzene (DNCB) and all other chemicals were purchased from Sigma Chemical Co. (St. Louis, MO). Iscove’s modified Dulbecco’s medium (IMDM) fetal bovine serum (FBS), penicillin, and streptomycin were obtained from Life Technologies Inc. (Grand Island, NY). The enzyme immunoassay (EIA) kits for TNF-α, IL-6, and IL-1β were obtained from R&D Systems (Minneapolis, MN, USA). SYBR Premix Ex Taq was purchased from Takara (Shuzo, Shiga, Japan). TNF-α, IL-6, IL-1β, and GAPDH oligonucleotide primers were purchased from Bioneer (Daejeon, Chungbuk, South Korea). SB203580 was purchased from Sigma Chemical Co. (St. Louis, MO).

### Cell culture and sample treatment

Human mast-cells-1 (HMC-1) were kindly provided by Prof. Jae-Young Um (Kyung Hee University, South Korea) and were grown at 37 °C in IMDM supplemented with 10% FBS, penicillin (100 U/mL), and streptomycin (100 μg/mL) in a humidified atmosphere of 5% CO_2_. Cells were treated with roxatidine at concentrations of 6.25, 12.5, and 25 μM, or with SB203580 (40 μM), and then stimulated with 20 nM of PMA and 1 μM of A23187 (PMACI) for the indicated time. Various concentrations of test compounds dissolved in DMSO were added together with PMACI. Cells were either treated with 0.05% DMSO as vehicle control.

### Compound 48/80-induced anaphylactic shock model

ICR male mice (6 weeks old) were obtained from Raon Bio Co. (Yongin, Gyeonggi, Republic of Korea) and maintained under constant conditions (temperature of 20–25 °C, humidity of 40–60%, 12 h light/dark cycle). The mice were randomly assigned to one of four groups (n = 6 per group). The ICR mice were injected intraperitoneally with PBS or compound 48/80 (8 mg/kg dissolved in PBS). Roxatidine or disodium cromoglycate (DSCG, Sigma-Aldrich, MO, USA) were dissolved in saline and orally administered at doses of 25 mg/kg DSCG and 20 mg/kg roxatidine for 1 h before the compound 48/80 injection. Mortality was monitored for 1 h after induction of anaphylactic shock. After the mortality test, blood was taken from the heart of each mouse to measure serum histamine content. Blood was clotted for 1 h at room temperature and centrifuged for 20 min at 3,000× *g* at 4 °C to obtain serum for cytokine production. All procedures were conducted in accordance with university guidelines and approved by the ethical committee for Animal Care and the Use of Laboratory Animals, Oriental Medicine, Sang-ji University (approval documents: 2015–12).

### Induction of contact hypersensitivity (CHS)

C57BL/6 male mice (6 weeks old) were sensitized with 1% DNCB in acetone-olive oil (4:1) on both ears (20 μl to each side of the ear). Roxatidine or dexamethasone (Sigma-Aldrich, MO, USA) were dissolved in saline and orally administered at doses of 3 mg/kg dexamethasone and 20 mg/kg roxatidine for 1 h before the CHS induction. 24 h later, mice were euthanized and a disk of ear tissue was removed from both ears using a 6 mm biopsy punch, then each of ear tissue was weighed. Relative ear swelling was measured as the increases compared to prechallenge ear weight. In addition, to determined numbers of mast cells, collected ear tissues were fixed in 10% buffered formalin, embedded in paraffin, sectioned into 4 μm slices, and stained with toluidine blue for evaluation of mast cell infiltration. Mast cell counts of 20 thin-sections from each specimen were averaged to obtain mast cell density per square millimeter.

To analyze the migration of FITC-bearing cutaneous DCs from skin to draining LNs, mice were sensitized with a total of 100 μl of 2% FITC isomer-I in a vehicle consisting of acetone-dibutylphthalate (1:1) administered to the shaved back. 48 h later, both axillary LNs were isolated and numbers of migrated FITC-bearing DCs were analyzed by flow cytometry (Beckman Coulter, CA, USA).

### Cytokine assays

Culture media and tissues of liver and ear were collected and stored at −70 °C. The production levels of TNF-α, IL-6, and IL-1β were measured by EIA kits (R&D Systems, MN, USA) according to the manufacturer’s instructions.

### Western blot analysis

Segments of cells or liver tissue were suspended in PRO-PREP™ protein extraction solution (Intron Biotechnology, Seoul, South Korea) and incubated for 20 min at 4 °C. Cell debris was removed by micro-centrifugation, followed by quick freezing of the supernatant. The protein concentration was determined using the Bio-Rad protein assay reagent according to the manufacturer’s instructions. Cellular protein from treated and untreated cell extracts was electro-blotted onto a polyvinylidene fluoride (PVDF) membrane following separation on a 10–12% sodium dodecyl sulfate (SDS) polyacrylamide gel electrophoresis. The immune blot was incubated for 1 h with blocking solution (5% skim milk) at room temperature, followed by incubation overnight with a primary antibody at 4 °C. Blots were washed three times with Tween 20/Tris-buffered saline (T/TBS) and incubated with a 1:1,000 dilution of horseradish peroxidase-conjugated secondary antibody for 2 h at room temperature. Blots were again washed three times with T/TBS, and then developed by enhanced chemiluminescence (GE healthcare, WI, USA).

### Quantitative real-time PCR analysis

Total RNA was isolated from cells or liver tissue using a Trizol reagent (Invitrogen, Carlsbad, CA, USA). cDNA was obtained using isolated total RNA (2 μg), d(T)16 primer, and AMV reverse transcriptase. Relative gene expression was quantified by quantitative real-time PCR (Real Time PCR System 7500, applied bio systems, Foster, CA) with SYBR Premix Ex Taq. The oligonucleotide primers for *TNF*-*α* were GCTGGAGAAGGGTGACCGAC (forward) and GTTCGTCCTCCTCACAGGGC (reverse); for *IL*-*6*: ATTCCGGGAACGAAAGAGAA (forward) and TCTTCTCCTGGGGGTACTGG (reverse); for *IL*-*1β*: TGGACCTCTGCCCTCTGGAT (forward) and GGCAGGGAACCAGCATCTTC (reverse); for indicate target gene *GAPDH*: CTCCTCCACCTTTGACGCTG (forward) and CTCTTGTGCTCTTGCTGGGG (reverse). The size of the synthesized cDNAs was 200 bp. The results are expressed as the ratio of optimal density to *GAPDH*.

### Preparation of nuclear protein extraction

HMC-1 were plated in 60 mm dishes (1 × 10^6 ^cells/mL) and treated with roxatidine (6.25,12.5, and 25 μM) for 1 h. They were then stimulated with PMACI for 30 min, washed once with PBS, harvested into 1 mL of cold PBS, and pelleted by centrifugation. Nuclear extracts were prepared as described previously^49^. Cell pellets were re-suspended in hypotonic buffer (10 mM HEPES, pH 7.9, 1.5 mM MgCl_2_, 10 mM KCl, 0.2 mM PMSF, 0.5 mM DTT, 10 mg/mL aprotinin) and incubated on ice for 20 min. Cells were then lysed by adding 0.1% Nonidet P-40 and vortexing vigorously for 10 s. Nuclei were pelleted by centrifugation at 12,000× g for 1 min at 4 °C and re-suspended in high salt buffer (20 mM HEPES, pH 7.9, 25% glycerol, 400 mM KCl, 1.5 mM MgCl_2_, 0.2 mM EDTA, 0.5 mM DTT, 1 mM NaF, 1 mM sodium orthovanadate).

### Histamine assay

Serum levels of histamine in animals were measured using commercially available EIA kits (Enzo Life Sciences, USA) according to the manufacturer’s recommendations.

### Statistical analysis

Data are expressed as the mean ± standard deviation (SD) of triplicate experiments. Statistically significant values were compared using ANOVA and Dunnett’s post hoc test, and P-values of less than 0.05 were considered statistically significant differences.

## Additional Information

**How to cite this article**: Lee, M.-H. *et al*. Roxatidine attenuates mast cell-mediated allergic inflammation via inhibition of NF-κB and p38 MAPK activation. *Sci. Rep.* **7**, 41721; doi: 10.1038/srep41721 (2017).

**Publisher's note:** Springer Nature remains neutral with regard to jurisdictional claims in published maps and institutional affiliations.

## Supplementary Material

Supplementary Data
