# Supplementary material for: Roxatidine attenuates mast cell-mediated allergic inflammation via inhibition of NF-κB and p38 MAPK activation
Source: Sci Rep. 2017 Jan 31;7:41721. doi: 10.1038/srep41721 (PMC5282503; doi:10.1038/srep41721)
Supplement: Supplementary Data [file srep41721-s1.docx]

**Roxatidine attenuates mast cell-mediated allergic inflammation via inhibition of NF-κB and p38 MAPK activation**

Min-Ho Lee^1^, Na Young Lee^2^, Kyung-Sook Chung^1^, Se-Yun Cheon^2^, Kyung-Tae Lee^3^, and Hyo-Jin An^2*^

^1^Catholic Precision Medicine Research Center, College of Medicine, The Catholic University of Korea, 222, Banpo-daero, Seocho-gu, Seoul, 06591, Republic of Korea

^2^Department of Pharmacology, College of Korean Medicine, Sangji University, Gangwon-do 220-702, Republic of Korea

^3^Department of Pharmaceutical Biochemistry, Kyung Hee University, Seoul 130-701, Republic of Korea

**Supplementary Data**


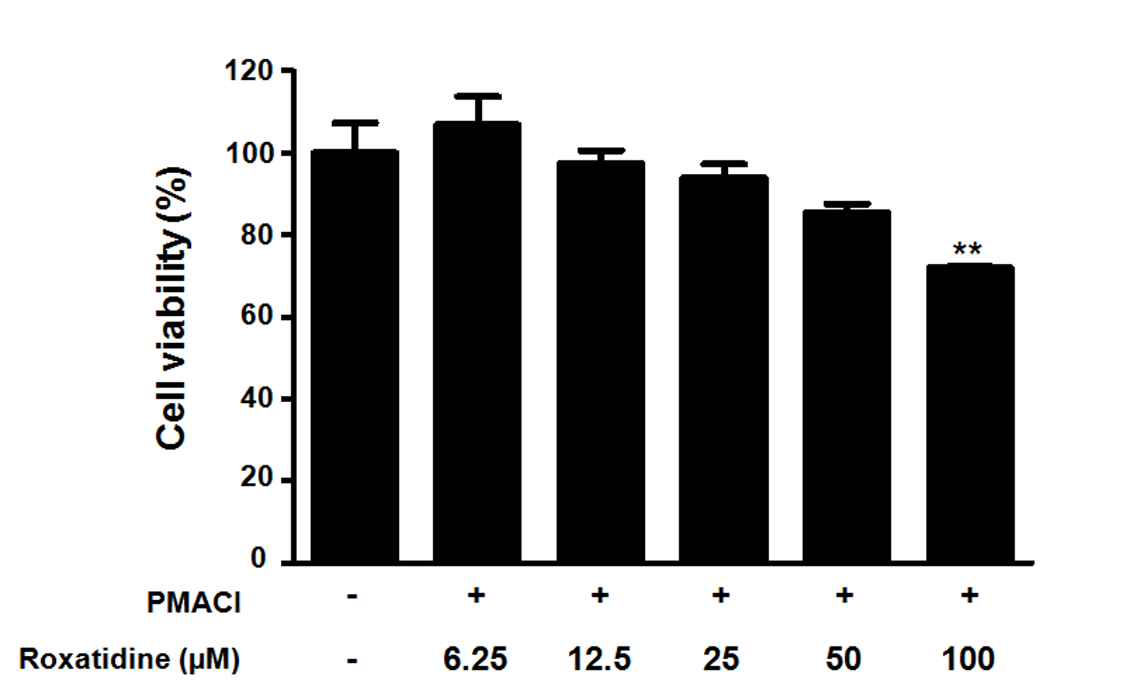


**Supplementary Figure1. Effect of roxatidine on cell viability in HMC-1.** Cells were treated with roxatidine (0 -100 µM) in the present or absent of PMACI for 24 h, and cell viabilities were determined by MTT assay. Data are presented as the means ± SDs of three independent experiments. ^***^*p*< 0.001 compared with the Control group; the significances of differences between treated groups were evaluated using ANOVA and Dunnett’s post hoctest.

Values represent mean ± S.D. of three independent experiments. ^**^*p < 0.01* vs. PMACI-treated group.
